# Proposal of the CAD System for Melanoma Detection Using Reconfigurable Computing

**DOI:** 10.3390/s20113168

**Published:** 2020-06-03

**Authors:** Wysterlânya K. P. Barros, Daniel S. Morais, Felipe F. Lopes, Matheus F. Torquato, Raquel de M. Barbosa, Marcelo A. C. Fernandes

**Affiliations:** 1 Laboratory of Machine Learning and Intelligent Instrumentation, Federal University of Rio Grande do Norte, Natal, RN 59078-970, Brazil; w.kyury@live.com (W.K.P.B.); daniel.moraisrn@gmail.com (D.S.M.); lopesffernandes@gmail.com (F.F.L.); 2 College of Engineering, Swansea University, Swansea, Wales SA2 8PP, UK; m.f.torquato@swansea.ac.uk; 3 Laboratory of Drug Development, Department of Pharmacy, Federal University of Rio Grande do Norte, Natal, RN 59078-970, Brazil; raquelmb@mit.edu; 4 Department of Computer and Automation Engineering, Federal University of Rio Grande do Norte, Natal, RN 59078-970, Brazil

**Keywords:** artificial neural networks, digital image processing, melanoma detection

## Abstract

This work proposes dedicated hardware to real-time cancer detection using Field-Programmable Gate Arrays (FPGA). The presented hardware combines a Multilayer Perceptron (MLP) Artificial Neural Networks (ANN) with Digital Image Processing (DIP) techniques. The DIP techniques are used to extract the features from the analyzed skin, and the MLP classifies the lesion into melanoma or non-melanoma. The classification results are validated with an open-access database. Finally, analysis regarding execution time, hardware resources usage, and power consumption are performed. The results obtained through this analysis are then compared to an equivalent software implementation embedded in an ARM A9 microprocessor.

## 1. Introduction

In recent years, there has been a notable increase in the development of technologies applied to the health area, bringing several benefits to professionals responsible for giving diagnosis. One such technology is Computer-Aided Diagnosis (CAD) which is applied on detecting and diagnosing various kinds of clinical conditions by using different varieties of medical imaging. These systems aim to assist with medical decisions about treatment and prognosis and improve the patient’s quality of life [1].

CAD systems often present a high computational cost, since many of these are based on the use of machine learning algorithms and digital image processing that can be computationally costly. An alternative to provide better performance in terms of execution time and power consumption is the implementation of these algorithms in a Field Programmable Gate Array (FPGA), a reconfigurable hardware consisting of several configurable logic blocks and programmable interconnects, which can be designed to implement a desired circuit.

According to [2], the use of FPGAs provides the system with fast execution and low power consumption, allowing the development of real-time embedded systems. The use of FPGAs in the development of CAD systems is the object of several works in the literature [3,4,5,6,7,8].

The work of [9] proposes an embedded system for recording real-time multimodal images applied to non-invasive tracking of skin cancer. The work presents the development of a hybrid architecture of hardware and software, implemented in a Xilinx Z-7010 SoC device. The hardware implements the detection and extraction of image characteristics in the visible and infrared spectrum, while the software estimates the geometric transformation that maps one image in the other and then applies the transformation to the frames of the infrared images.

The work proposed by [10] presents the implementation of an on-chip Multilayer Perceptron (MLP) to ensure the safety of electronic devices used in the treatment of diabetes. These devices are usually insulin pumps, which can have its system invaded and send false insulin dosing commands. The implemented MLP architecture has two hidden layers, the first with seven neurons and the second with four and an output layer with only one output neuron. In the neurons of the hidden layers, the hyperbolic tangent activation function was used, while the sigmoid function was used in the neurons of the output layer.

Only one research group has been found in the literature that proposes a melanoma detection system using FPGA. This group has several works in the literature reporting the advances of this research of melanomas. In all works, the target FPGA was the Xilinx XC7Z020CLG484-1 from the Zynq-7000 ZC702 Evaluation Board. The implementations proposed by this research group were developed in C/C ++ using the UltraFast High-Level Synthesis (HLS) tool available in the Xilinx Vivado Design Suite, and a Support Vector Machine (SVM) is designed as an HLS Intellectual Property (IP).

The first paper presented by this group in the literature, in [11], proposed the implementation of a hardware/software co-design, implementing in hardware only the scalar product present in the SVM classifier function and all other necessary calculations were performed in software. In this work, the test data are transmitted using a streamming interface with an Direct Memory Access (DMA) IP. Then, the hardware design was extended to implement the full function of the SVM classifier, as presented in [12]. Another similar project was proposed in [13], which used embedded block RAM (BRAM) interfaces to pass required data instead of using the stream interface with the DMA IP. The work presented in [14] sought to simplify previously designed IPs to reduce hardware area, power and cost for project extension, as well as to improve rating performance. It proposed a reconfigurable hardware system for the implementation of an adaptive, flexible, and a scalable embedded cascade SVM classifier system.

The most recent article from this group is presented in [15]. It proposes three SVM models generated from the available feature set training, using 356 instances with 27 features each. The first model was generated using the complete original data set, employing in this model 346 Support Vectors (SVs). Next, scaling and normalization techniques were applied to the original dataset, which generated a second model with 248 SVs and achieved higher classification accuracy. The third, smaller-scale model was implemented to be used as a case study for performance validation by running on a Zynq SoC, while the other two models were validated using only simulation results. This model consists of 61 SVs, generated using only part of the data set (144 instances) normalized in the training phase. Several versions of these models were implemented using different optimization techniques through the available optimization directives of the Vivado HLS tool. This article presents several results which are later presented for comparison with the architecture proposed here.

In this scenario, the objective of this work is the hardware implementation of a skin cancer detection system, using digital image processing techniques and a multilayer perceptron artificial neural network. The hardware developed for the DIP techniques is responsible for extracting the desired descriptors from the skin nevus image, while the hardware developed for the feedforward phase (inference step) of the MLP is in charge of performing the classification of the skin signal as melanoma or non-melanoma based on the descriptors extracted by the DIP, using the weights previously obtained by training the network in a software implementation. In addition, it is intended to achieve high speed performance and low energy consumption. Results regarding hardware resources occupation, runtime and power consumption are detailed and presented for an Intel Cyclone V FPGA SE 5CSEBA6U23I7. Intel Cyclone V FPGA SE 5CSEBA6U23I7 has 41,910 ALMs, 166,036 registers, 5570 M10K memory blocks, and 224 DSP blocks.

The paper is structured as follows: Section 2 presents a skin cancer detection technique developed based on existing methods and its software implementation used to define the parameters of the neural network through the training phase. Section 3 presents the details of the hardware architecture, describing the various modules and submodules used to implement the system. In Section 4, the results of the proposed hardware validation and the implementation synthesis are presented. Finally, Section 5 presents the final considerations about the manuscript.

## 2. Skin Cancer Detection Technique

With digital image processing, nine descriptors are extracted from a nevus image. These descriptors are then forwarded to the MLP, which is trained to classify the images into two distinct classes: melanoma and non-melanoma. After the network training, its validation is performed using a public database of skin cancer images that was previously diagnosed by specialists.

### 2.1. Technical Overview

**Figure 1:**
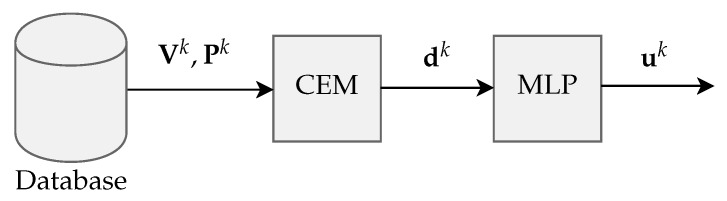
General structure of the proposed detection scheme.

Figure 1 represents the proposed detection structure, in which the classification of skin nevi is performed using a database of dermatoscopic images.

The N×M pixels image from the database $V^{k}$ is expressed as

(1) $$V^{k}=\left[\begin{array}{ccccc} v_{0,0}^{k} & \cdots & v_{0,j}^{k} & \cdots & v_{0,M-1}^{k}\\ \vdots & \vdots & \vdots & \vdots & \vdots \\ v_{i,0}^{k} & \cdots & v_{i,j}^{k} & \cdots & v_{i,M-1}^{k}\\ \vdots & \vdots & \vdots & \ddots & \vdots \\ v_{N-1,0}^{k} & \cdots & v_{N-1,j}^{k} & \cdots & v_{N-1,M-1}^{k} \end{array}\right],$$

where $v_{ij}^{k}$ corresponds to a pixel of *b*-bit pixel of the *k*-th image from the database. A binary image, $P^{k}$, is used as a mask to extract the region of interest from the original image. The $P^{k}$ image is defined as

(2) $$P^{k}=\left[\begin{array}{ccccc} p_{0,0}^{k} & \cdots & p_{0,j}^{k} & \cdots & p_{0,M-1}^{k}\\ \vdots & \vdots & \vdots & \vdots & \vdots \\ p_{i,0}^{k} & \cdots & p_{i,j}^{k} & \cdots & p_{i,M-1}^{k}\\ \vdots & \vdots & \vdots & \ddots & \vdots \\ p_{N-1,0}^{k} & \cdots & p_{N-1,j}^{k} & \cdots & p_{N-1,M-1}^{k} \end{array}\right]$$

where $p_{ij}^{k}$ corresponds to a pixel of the *k*-th binary image from the database.

With the mask $P^{k}$, the original image $V^{k}$ passes through a block referred as Character Extractor Module (CEM), which extracts from the image nine descriptors expressed as $d^{k}=\left[d_{0}^{k},\ldots ,d_{8}^{k}\right]$. This set of descriptors is then processed by an MLP-BP artificial neural network that contains nine inputs, three layers of neurons with two hidden layers, and the output layer with two outputs which are expressed as $u^{k}=\left[u_{0}^{k}\;u_{1}^{k}\right]$.

**Figure 2:**
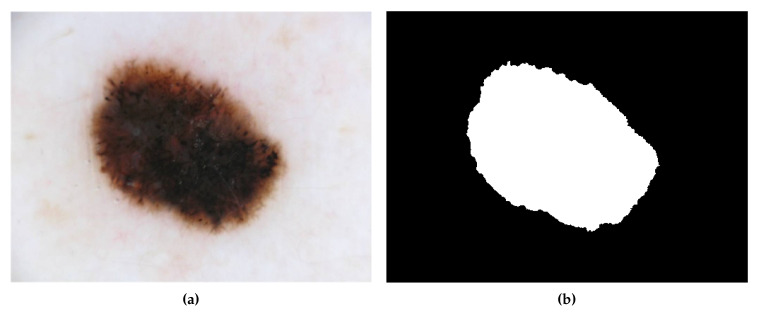
Database sample. (**a**) Original image; (**b**) Binary image.

A database containing 200 dermatoscopic images provided by the ADDI Project [16] was used. The images have a resolution of 768 × 560 pixels and magnification of 20×. In this work, the resolution was halved. Masks for each image containing the delimitation of the Nevus region are also available. The images were diagnosed by specialists and were divided into 160 non-melanoma and 40 melanomas. Figure 2b shows the binary image $P^{k}$ correspondent to the sample shown in Figure 2a.

### 2.2. Character Extractor Module (CEM)

In the literature, there are several attempts to simplify the dermoscopic approach to diagnose benign melanocytic lesions and melanomas, as presented in [17], such as the ABCD rule, the Menzies method, and the 7-point checklist.

The three approaches presented are for the recognition of melanoma based on dermatoscopic images, but there is also a rule for detection with the naked eye, which is called ABCDE. This rule is very similar to the ABCD rule and in it each letter indicates a characteristic of the signal to be analyzed, with A referring to asymmetry, B to edge, C to color, D to diameter and E to evolution [18,19,20].

Thus, based on these approaches, the work presented here defined a set of nine descriptors with mathematical representation: symmetry in *x*, symmetry in *y*, diameter, variance and mean in the *R* channel, variance and mean in the channel *G* and variance and mean in the channel *B*. These descriptors were represented by the variables, $d_{0},\ldots ,d_{8}$, respectively. Thus, for a given image $V^{k}$, there is a vector $d^{k}=\left[d_{0}^{k},\ldots ,d_{8}^{k}\right]$ of descriptors.

#### 2.2.1. Symmetry Calculation

**Figure 3:**
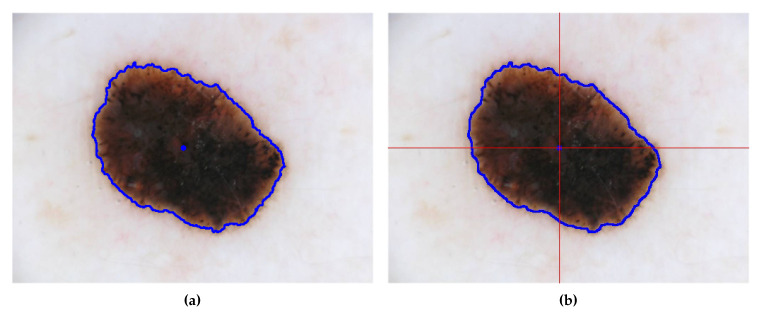
(**a**) Original image with the signal contour and center of mass represented by the blue dot; (**b**) Image divided in four quadrants with intersection of the axes in the center of mass.

The symmetry in *x* and *y* are represented by the descriptors $d_{0}^{k}$ and $d_{1}^{k}$. The calculation of these descriptors for a given image *k* are expressed as

(3) $$d_{0}^{k}=\left|\sum_{\begin{array}{c} i=0\\ i<c_{x}^{k} \end{array}}^{N-1}\sum_{j=0}^{M-1}p_{ij}^{k}-\sum_{\begin{array}{c} i=0\\ i\geq c_{x}^{k} \end{array}}^{N-1}\sum_{j=0}^{M-1}p_{ij}^{k}\right|$$

and

(4) $$d_{1}^{k}=\left|\sum_{i=0}^{N-1}\sum_{\begin{array}{c} j=0\\ j<c_{y}^{k} \end{array}}^{M-1}p_{ij}^{k}-\sum_{i=0}^{N-1}\sum_{\begin{array}{c} j=0\\ j\geq c_{y}^{k} \end{array}}^{M-1}p_{ij}^{k}\right|$$

where $c_{x}^{k}$ and $c_{y}^{k}$ are the center of mass values from the binary image, $P^{k}$. The center of mass is calculated by initially extracting the boundary points of the binary image, $P^{k}$, using an OpenCV library function, *findContours()* and later, using the result of this function as input to another OpenCV function, *moments()*, which returns the center of mass. Figure 3 shows the image divided in four quadrants with intersection of the axes in the center of mass.

#### 2.2.2. Diameter Calculation

**Figure 4:**
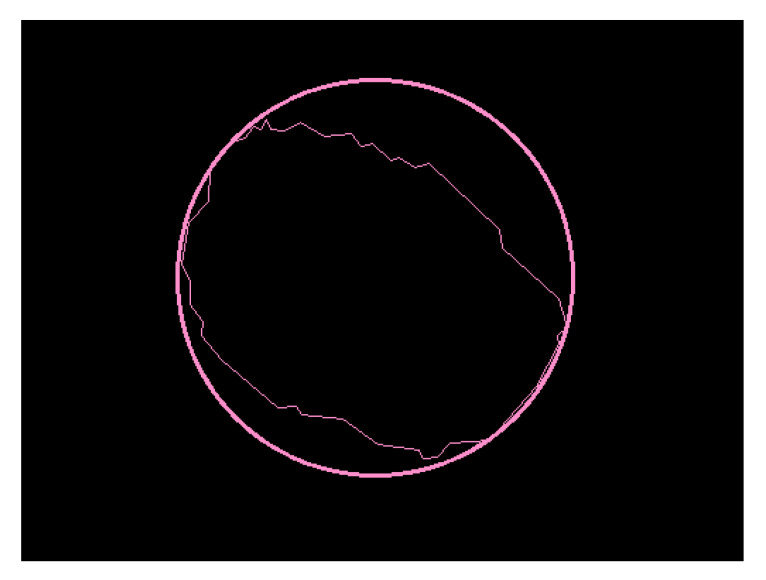
Circumference around the nevus.

The second descriptor is the diameter, represented by $d_{2}^{k}$. For the calculation of this descriptor, an OpenCV library function *minEnclosingCircle()* was used, which locates a circle of minimum area from the set of 2D points provided, which in this case are the points that form the contour of the binarized image. This function outputs the radius of the circle. Figure 4 shows the result of obtaining the diameter in the binarized image shown in Figure 2b.

#### 2.2.3. Calculation of Mean and Variance

The last descriptors are related to the color variation associated with the nevus and can be found through the mean and the variance in the RGB channels of the original image. The mean and variance of the R channel are represented by the descriptors $d_{3}^{k}$ and $d_{4}^{k}$, for channel G, the descriptors are $d_{5}^{k}$ and $d_{6}^{k}$ and for channel B the descriptors are $d_{7}^{k}$ e $d_{8}^{k}$. The calculation of each descriptor can be expressed as

(5) $$d_{3}^{k}=\frac{1}{L}\sum_{i=0}^{N-1}\sum_{j=0}^{M-1}R\left(v_{ij}^{k}\right)\times p_{ij}^{k}$$

and

(6) $$d_{4}^{k}=\frac{1}{L}\sum_{i=0}^{N-1}\sum_{j=0}^{M-1}{\left(R\left(v_{ij}^{k}\right)-d_{3}^{k}\right)}^{2}\times p_{ij}^{k}$$

in which *L* is the number of pixels equal to 1 in the binarized matrix, $P^{k}$, and $R(v_{ij}^{k})$ is the pixel value $v_{ij}^{k}$ in channel *R*. Following the same idea, the value of the descriptors for the other channels can be expressed as

(7) $$d_{5}^{k}=\frac{1}{L}\sum_{i=0}^{N-1}\sum_{j=0}^{M-1}G\left(v_{ij}^{k}\right)\times p_{ij}^{k},$$

(8) $$d_{6}^{k}=\frac{1}{L}\sum_{i=0}^{N-1}\sum_{j=0}^{M-1}{\left(G\left(v_{ij}^{k}\right)-d_{5}^{k}\right)}^{2}\times p_{ij}^{k},$$

(9) $$d_{7}^{k}=\frac{1}{L}\sum_{i=0}^{N-1}\sum_{j=0}^{M-1}B\left(v_{ij}^{k}\right)\times p_{ij}^{k},$$

and

(10) $$d_{8}^{k}=\frac{1}{L}\sum_{i=0}^{N-1}\sum_{j=0}^{M-1}{\left(B\left(v_{ij}^{k}\right)-d_{7}^{k}\right)}^{2}\times p_{ij}^{k}.$$

### 2.3. MLP

**Figure 5:**
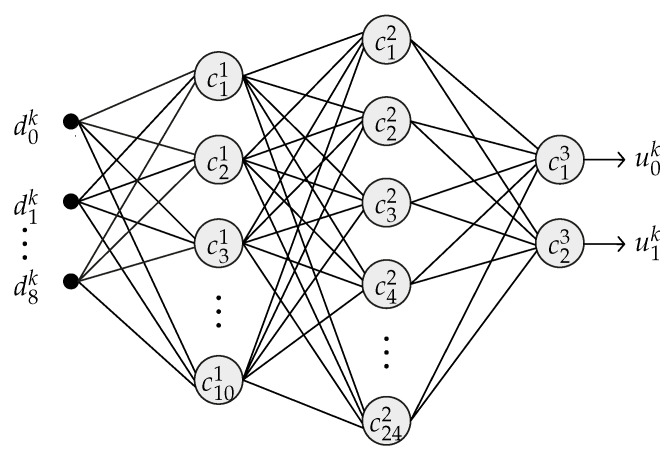
General structure of the proposed MLP.

In order to solve the melanoma classification, the work proposed here uses an MLP-BP with nine inputs (the descriptors), two hidden layers, and two outputs. The proposed network architecture contains 10 neurons in the first hidden layer, represented by $c_{1..10}^{1}$, and 24 neurons in the second, represented by $c_{1..24}^{2}$. The activation function used in all the neurons of the network was the sigmoid function. Figure 5 shows the described network structure.

The outputs of the network, $u^{k}=\left[u_{0}^{k}\;u_{1}^{k}\right]$, classify the image as melanoma or non-melanoma, being $u_{0}^{k}$ for melanoma and $u_{1}^{k}$ a non-melanoma. When $u^{k}=\left[0\;1\right]$, the classified signal is non-melanoma and when $u^{k}=\left[1\;0\right]$ indicates that the nevus is melanoma.

All of the image descriptors $d^{k}$ are normalized between 0 and 1 in order to to improve the convergence of the network. The normalization of each descriptor was performed by dividing all elements of it by the highest value within each descriptor, taking into account 200 images of the database.

**Figure 6:**
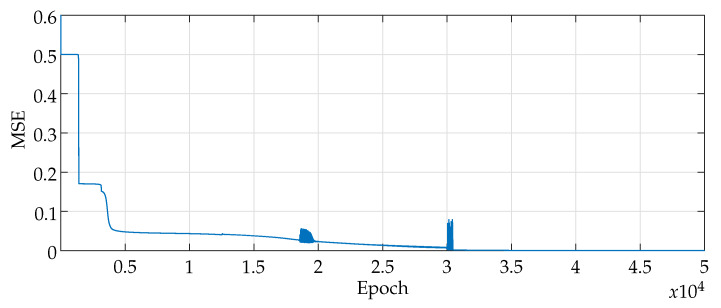
MLP Learning Curve.

For the training and validation of the network, all the images made available by the database PH ${}^{2}$*Database* were used. During the network training phase, 170 randomly selected images from the bank were used, divided into 138 non-melanomas and 32 melanomas. The MLP converged into a $10^{-6}$ error, as shown in Figure 6, which illustrates the network mean squared error by the number of epochs. During training, the neural network weights are adjusted. The final network model employs the weights wherewith they obtained the lowest error.

**Figure 7:**
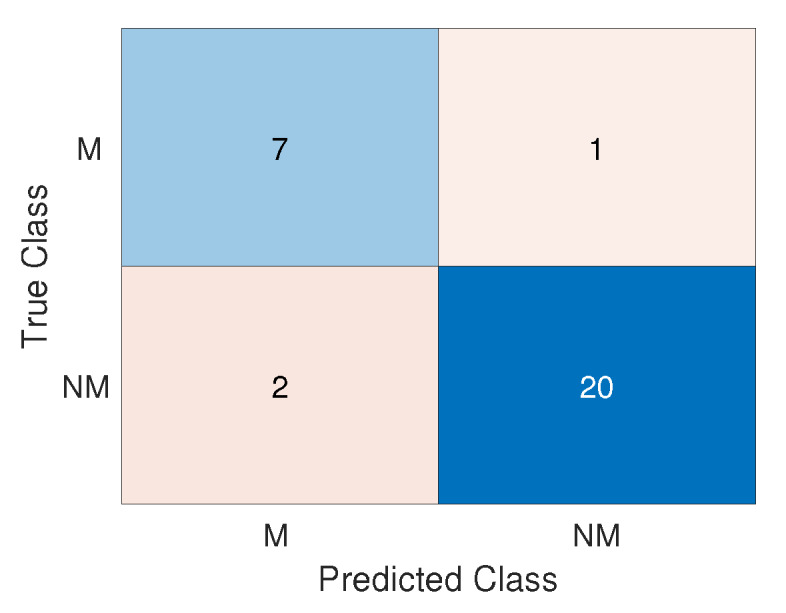
Confusion matrix of validation results.

After defining the final model weights in the training phase, the model validation is performed using 30 images, which were divided into 22 non-melanomas and 8 melanomas. According to the result of the validation data, only three errors were obtained, one false negative, and two false positives. Figure 7 illustrates the validation results in the confusion matrix. The actual classes are arranged in rows while the predicted classes are arranged in columns. The correct classified nevis are represented on the main diagonal of the matrix and the incorrect on the antidiagonal. The classes are represented by the acronyms M and NM, which respectively indicate melanoma and non-melanoma.

In order to evaluate the performance of the proposed technique, the work proposed here presents three measures of common use in several works with similar classification problems [21,22,23,24], which are: accuracy, specificity, and sensitivity [25]. Accuracy is the model’s ability to correctly classify cases of melanoma and non-melanoma, being the number of correct classifications divided by the number of all data classified. Occurring 27 correct diagnosis in 30 cases, the technique obtained an accuracy of 90%. Specificity is the proportion of non-melanoma correctly identified by classifier, so with 20 non-melanomas correctly recognized in 22 non-melanoma cases, a specificity of 90.9% was obtained. Finally, the sensitivity is the proportion of melanomas that are correctly identified by classifier, with seven melanomas correctly recognized in eight cases of melanoma, the sensitivity was 87.5%. Table 1 shows results obtained by the technique proposed here together with the ones found in the literature.

The equivalent hardware implementation of the proposed project to be described in the next section targeted designing an embedded system with the same classification results previously obtained but optimizing the performance and energy consumption.

## 3. Design Description

The hardware architecture was developed using fixed point number representation, with the values in the integer part ranging from 0 to 35 bits and in the fractional part ranging from 0 to 15 bits. The system’s inputs are represented in fixed point by 8 bits in the integer part and 0 in the fractional part, since the value of the RGB channels are unsigned integers ranging from 0 to 255. The image descriptors and the system outputs are represented with 0 bits in the integer part and 10 bits in the fractional part because they are unsigned values between 0 and 1.

**Figure 8:**
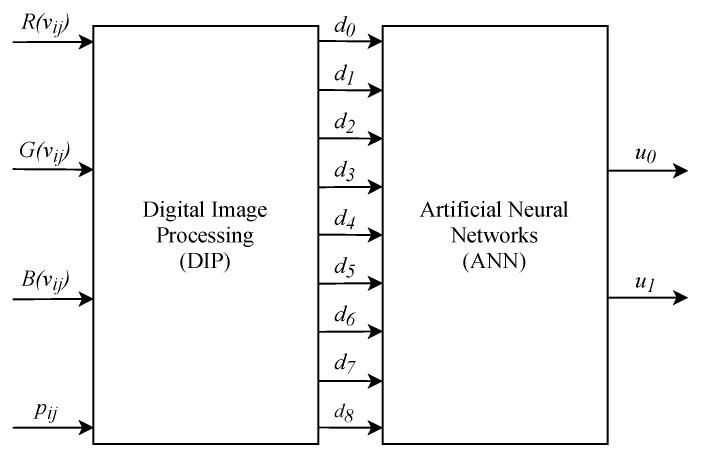
General hardware architecture.

Figure 8 presents a general hardware architecture. This figure shows the two main modules of the system, the module of Digital Image Processing techniques and the Artificial Neural Network. The inputs of this architecture are the pixels of the image, ***V***, to be classified and the image mask, ***P***, which is provided by the database used.

The input $R(v_{ij})$ refers to the intensity of the pixel in the channel R of the image ***V*** in the i-th line and j-th column, the inputs $G(v_{ij})$ and $B(v_{ij})$ follow the same idea, referring respectively to the intensity of the pixel in channel G and B. The input $p_{ij}$ is the pixel value of the binarized image ***P*** in the i-th row and j-th column, in which the pixels of the region of interest of the nevus are represented by 1 and the pixels of the background by 0. The output of the DIP module are the nine descriptors extracted from the nevus image, represented by $d_{0}$, $d_{1}$, …, $d_{8}$. These descriptors are the inputs of the ANN module, responsible for performing the image classification. The outputs of this module indicate the result of the classification, $u_{0}$ and $u_{1}$.

### 3.1. Digital Image Processing Module (DPIM)

The Digital Image Processing Module (DPIM) aims to perform the necessary operations on the pixels of the input image, ***V***, to obtain the descriptors, $d_{0}$, $d_{1}$, …, $d_{8}$. In this module, the technique of Stream Processing was used, requiring two image scans for the extraction of all the descriptors, since there are calculations that require values obtained only at the end of the first complete scan of the image. The image input was performed pixel by pixel, starting with the first line from left to right. This module has five main submodules, which are intended for: the application of the mask, calculation of symmetry, calculation of the diameter, calculation of the mean, and calculation of the variance.

**Figure 9:**
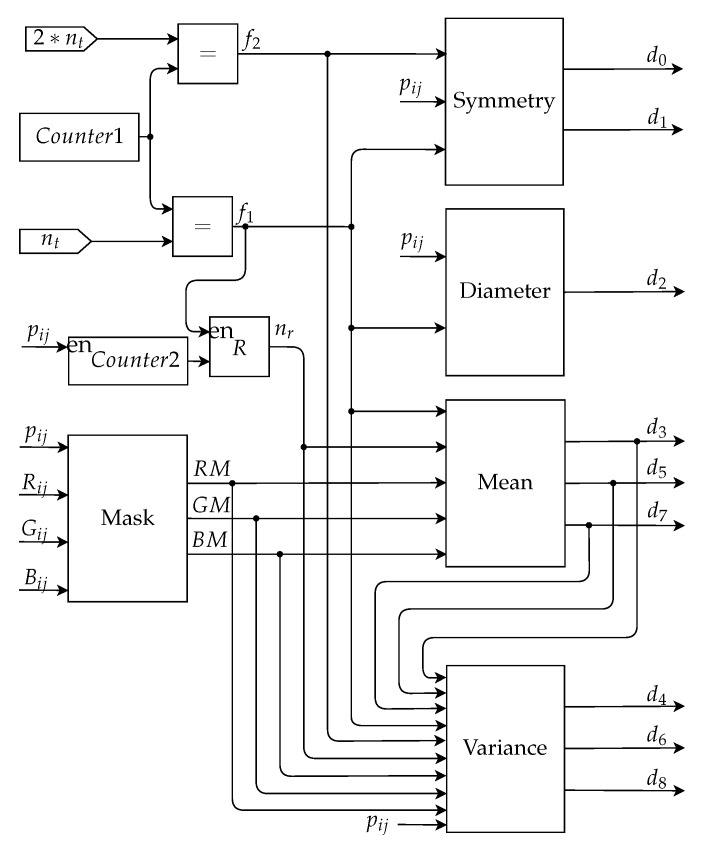
Digital image processing module architecture.

Figure 9 shows the general architecture of DPIM, with all its submodules. In this figure, $n_{t}$ represents a constant with a value equal to the total number of pixels of the analyzed image of the nevus. Some submodules have as input the variables represented in the figure as $n_{r}$, $f_{1}$ and $f_{2}$. The variable $n_{r}$ represents the number of pixels in the region of interest, this value is obtained through a counter, *Counter2*, which is enabled by the pixels of the binary image equal to 1. The value of this counter is stored in a register after the first scan of the image. The boolean variable $f_{1}$ indicates the end of the first scan of the image and the boolean variable $f_{2}$ indicates the end of the second scan of the image, when they assume a value of 1. The counter block, *Counter1*, has a maximum value equal to $2\times n_{t}$, and the counter block, *Counter2*, has a maximum value equal to $n_{t}$.

#### 3.1.1. Mask Application Submodule

The Mask Application Submodule is responsible for applying the binary mask, ***P***, on each channel of the original image ***V***. The mask used is provided by the database.

**Figure 10:**
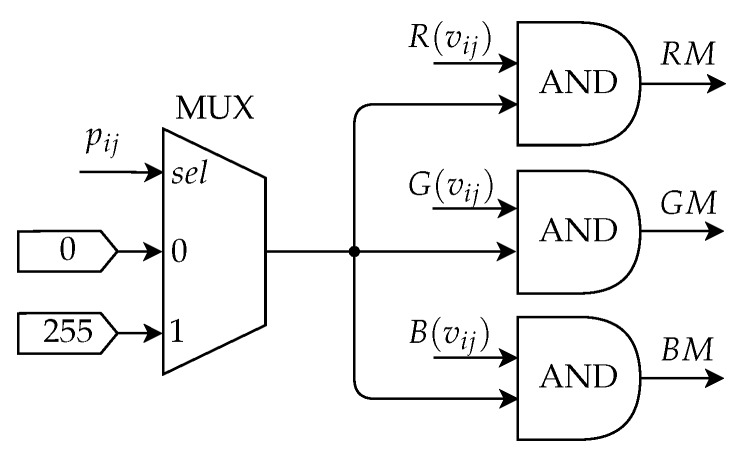
Architecture of the Mask Application Submodule.

Figure 10 shows the architecture of the Mask Application Submodule. The application of the mask on each channel is performed using an AND logic gate with eight bits in the integer part. The pixels of the binarized image with a value of 1 are converted to 255 by a multiplexer (MUX). Thus, after the MUX, the binarized image inputs equal to zero are represented by the binary value $00000000_{2}$ and equal to one by $11111111_{2}$. Thereby, after the AND operation, the pixels of the image within the region of interest have their value equal to the value of the original image, while the others representing the background have their value equal to zero. The RM, GM, and BM outputs are the value of the pixel intensity in each channel after the mask is applied.

#### 3.1.2. Symmetry Calculation Submodule

The Symmetry Calculation Submodule is responsible for calculating the values of the descriptors $d_{0}$ and $d_{1}$. For this, it is necessary to first determine the position of the center of mass of the nevus, $c_{x}$ and $c_{y}$. The calculation of the center of mass in software was performed using functions of the OpenCV library. In the hardware implementation, this calculation was performed using the mask image, ***P***, being expressed as

(11) $$c_{x}=\frac{\sum_{i=0}^{N-1}\sum_{j=0}^{M-1}p_{ij}\cdot i}{\sum_{i=0}^{N-1}\sum_{j=0}^{M-1}p_{ij}}$$

and

(12) $$c_{y}=\frac{\sum_{i=0}^{N-1}\sum_{j=0}^{M-1}p_{ij}\cdot j}{\sum_{i=0}^{N-1}\sum_{j=0}^{M-1}p_{ij}}$$

where $c_{x}$ indicates the *x* coordinate of the center of mass and $c_{y}$ the *y* coordinate.

**Figure 11:**
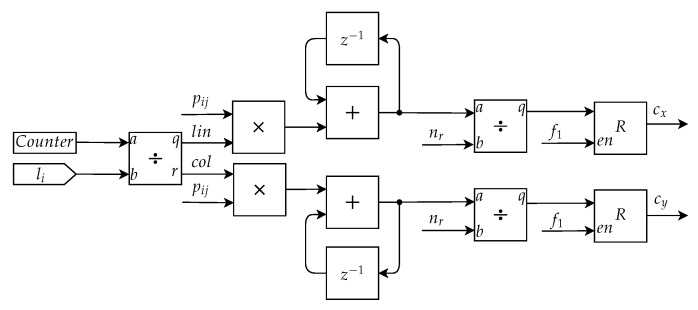
Architecture of the Symmetry Calculation Submodule responsible for calculating the center of mass.

Based on this mathematical representation, the calculation of the center of mass was implemented as shown in Figure 11. In this figure, $l_{i}$ represents a constant with a value equal to the width of the image. The counter indicates the number of pixels of the already read image. Dividing its value by the width of the image produces the value of the position of the input pixel. The variable *lin* indicates the line and the variable *col* the column where the pixel is positioned. Thereafter, there are two multipliers that perform the multiplication operations present in Equations (11) and (12), followed by the implementation of an accumulator with an adder block and a delayed feedback, which performs the operation of the double summation present in the equations. After the image is completely read, the value of each accumulator is divided by the number of pixels of the region of interest, the results obtained are $c_{x}$ and $c_{y}$. These values are stored in registers at the end.

**Figure 12:**
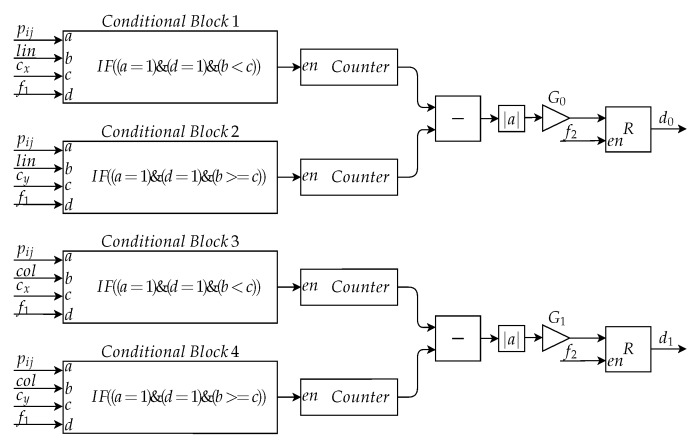
Architecture of the Symmetry Calculation Submodule to determine the symmetry.

After determining the center of mass, it is possible to calculate the symmetry. The proposed implementation for this calculation was based on Equations (3) and (4), and the designed architecture is shown in Figure 12.

Initially, there are four conditional blocks, each block equivalent to a double summation. Conditional Blocks 1 and 2 implement the first and second double summation of Equation (3), respectively, with the *i* of the equation represented by *lin*. Conditional Blocks 3 and 4 implement the first and second double summation of Equation (4), respectively, with the *j* of the equation represented by *col*. With the pixel of the binarized image equal to 1 and the logical expression of the conditional block being true, there is the increment of 1 to the counter, *counter*, presented after each conditional block. At the end of the second reading of the image, the difference between the values of the first and second counter in module is the result of Equation (3) and the modulus of the difference between the values of the third and fourth counter is the result of Equation (4). These values are further multiplied by specific gains, $G_{0}$ and $G_{1}$, which normalize the value of the descriptors between 0 and 1. The result obtained after this are the descriptors $d_{0}$ and $d_{1}$, which are stored in a register.

#### 3.1.3. Diameter Calculation Submodule

**Figure 13:**
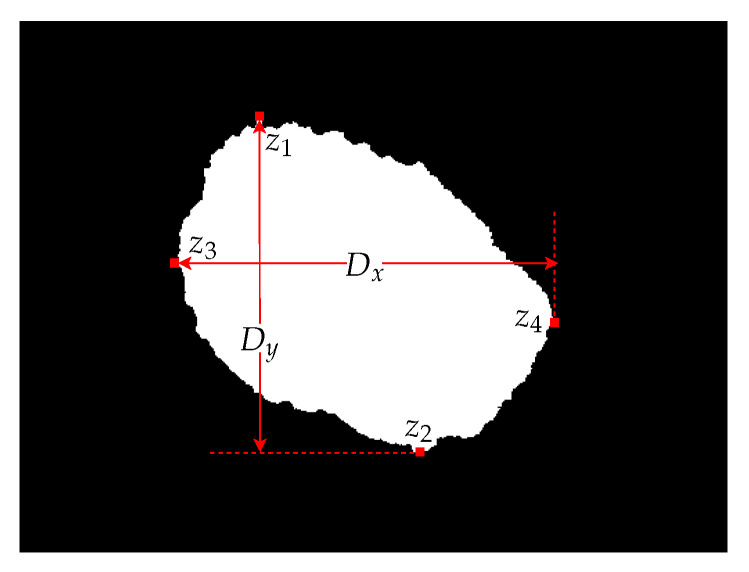
The four extremity pixels and the distances between them.

The Diameter Calculation Submodule has the function of calculating the value of the descriptor $d_{2}$. Initially, it is necessary find the most extreme points, $z_{1}$, $z_{2}$, $z_{3}$, and $z_{4}$, of the region of interest, based on the binarized image. After that, the distance, $D_{x}$ and $D_{y}$, between these pixels should be obtained. The largest distance is considered the diameter of the nevus. Figure 13 illustrates the dots and the distance between ends of Figure 2a.

**Figure 14:**
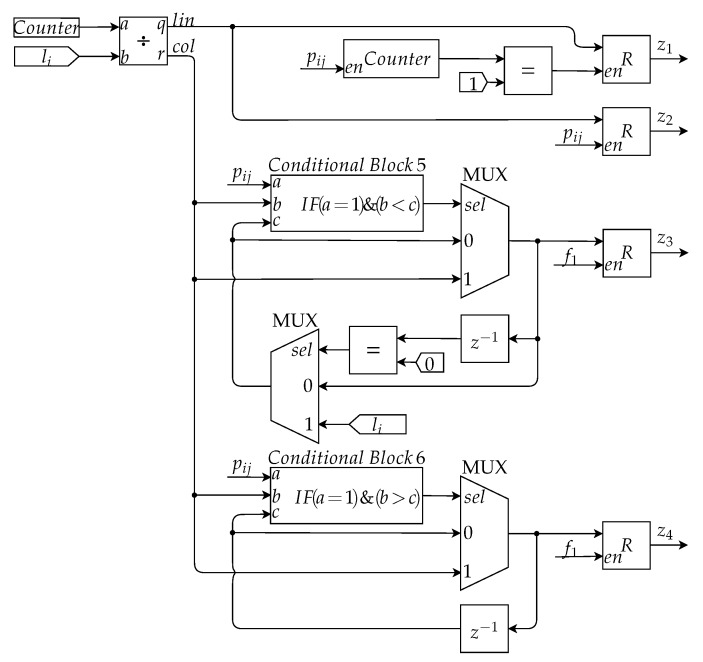
Architecture of the Diameter Calculation Submodule responsible for calculating the extremity pixels.

In Figure 14, the hardware architecture used to locate the extremity pixels in the region of interest is presented. Initially, there is a divisor block that receives its input from a counter and the constant $l_{i}$ in order to calculate the row and column of the input pixel. The first output of the architecture, $z_{1}$, indicates the line of the first pixel of the detected nevus which is the line in which $p_{ij}$ first assumes the value of 1. The second output, $z_{2}$, indicates the last line containing a pixel from the nevus previously detected. The value of $z_{2}$ is the last value stored in the register which is enabled by $p_{ij}$. The third output, $z_{3}$, indicates the first column to display a pixel from the nevus area, and this value is found by using a conditional block and a multiplexer.

The Conditional Block 5 (CB5) is responsible for comparing the column value of each pixel in the region of interest, input *b*, to select the lowest column value. During the image scan, the lowest value found is saved and applied to input *c* of CB5, for comparison with the other column values to be analyzed. The output of CB5 is true when the column value of the current pixel, input *b*, is less than the last found value, input *c*. The output of this block feeds the input selection of a multiplexer which for an input of 0 the value $z_{3}$ stored in the register continues the same and for an input of 1 this same value is updated to the value of the column of the current pixel $p_{ij}$. On top of that, another multiplexer is used so that, in the beginning of the calculation of $z_{3}$, the value of the *c* input is not equal to zero, but equal to the largest column value of the image.

Finally, the last output, $z_{4}$, indicates the last column in the region of interest. A conditional block and a multiplexer are also used to determine this pixel. Its operation is similar to the one previously presented for output $z_{3}$; however, it is unnecessary to use a second multiplexer since there is no problem if the initial value *c* is equal to zero. At the end of the first image scan, the selected values $z_{1}$–$z_{4}$ are stored in a register to, then, be forwarded to the diameter calculation architecture.

**Figure 15:**
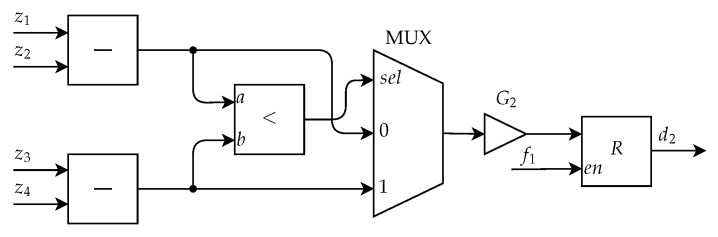
Architecture of the Diameter Calculation Submodule to determine the diameter.

Having found the extremity points of the nevus, it is then possible to calculate the diameter by the greatest distance among these points. The architecture implemented for this calculation is displayed in Figure 15. This module starts by calculating the difference between $z_{1}$ and $z_{2}$, which gives a distance on the *y*-axis and the difference between $z_{3}$ and $z_{4}$, which gives a distance on the *x*-axis. The result of these differences is compared and through a MUX the distance with greater value is selected, which is the approximate diameter of the nevus. At the end, the value found is multiplied by a gain, $G_{2}$, which normalizes the value of the descriptor between 0 and 1. The result obtained after this is the descriptor $d_{2}$, which is stored in a register.

#### 3.1.4. Mean Calculation Submodule

The Mean Calculation Submodule is responsible for calculating the value of the descriptors $d_{3}$, $d_{5}$, and $d_{7}$. This submodule calculates the average of the intensities of the pixels in the region of interest, using the image obtained after applying the mask. This implementation is based on the Equations (5), (7) and (9).

**Figure 16:**
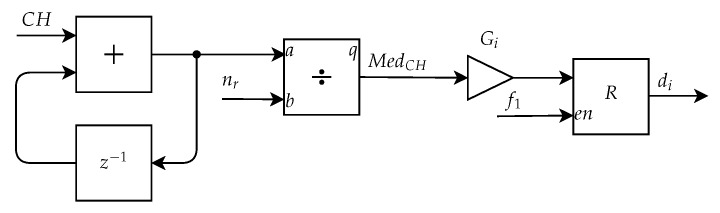
Architecture of the Mean Calculation Submodule.

Figure 16 presents the proposed implementation of this submodule, which applies the same processing in all of the channels of the image. This architecture uses as inputs the outputs from the Mask Application Submodule, RM, GM, and BM, represented in the figure by CH. Initially, there is an accumulator implemented by an addition block and a delay, equivalent to the double summation of Equations (5), (7) and (9). This accumulator sums for each channel all the values of the pixels in the region of interest and the final value of this accumulator is divided by the number of pixels from the nevus region. The result of each division, $Med_{R}$, $Med_{G}$, and $Med_{B}$, represented in the figure by $Med_{CH}$, is multiplied by a specific gain $G_{i}$, where i = [3, 5, 7]. The result obtained after this operation is the descriptor $d_{i}$, where i = [3, 5, 7], which are then stored into its respective registers.

#### 3.1.5. Variance Calculation Submodule

The Variance Calculation Submodule calculates the value of the descriptors $d_{4}$, $d_{6}$, and $d_{8}$, during the second image scan. This submodule calculates the variance of the pixels values from the region of interest using the outputs of the Mask Application Submodule and the Average Calculation Submodule. This implementation is based on the Equations (6), (8) and (10).

**Figure 17:**
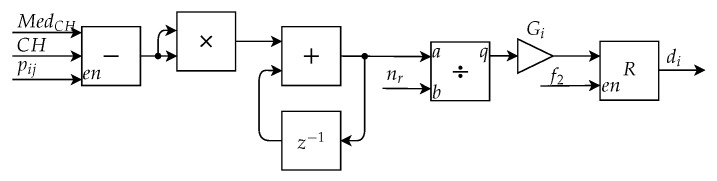
Architecture of variance.

In Figure 17, the architecture of the Variance Calculation Submodule is presented, which is also applied to each channel of the image. Initially, there is a block that calculates the difference between the channel average value and the pixel value from the Mask Application Submodule. This subtraction block is enabled when the pixel value in the binarized image $p_{ij}$ is equal to 1. In the following, there is a multiplier block with its two inputs fed by the output of the previous block; this is equivalent to a squared power operation as presented in Equations (6), (8) and (10). The output of this multiplier block is the input of an adder block with feedback, i.e., an accumulator. After the second image reading, the accumulator value is then divided by the number of pixels of the nevus. The result of each division is multiplied by a specific gain, $G_{i}$, where i = [4, 6, 8]. The results from this operation are the descriptors $d_{i}$, where i = [4, 6, 8], which are, finally, stored into its respective registers.

### 3.2. Artificial Neural Network Module (ANNM)

**Figure 18:**
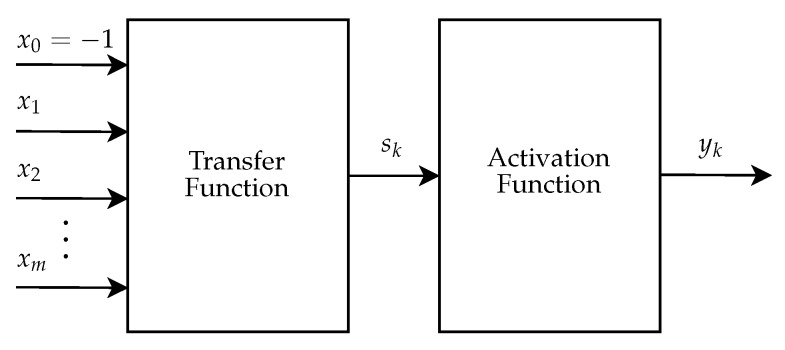
General architecture of the neuron.

The Artificial Neural Network Module (ANNM) aims to perform the image classification as melanoma or non-melanoma through an MLP neural network. This module was implemented with a full-parallel architecture. The general architecture of the ANNM is presented in Figure 5 and its entries are the nine descriptors extracted in the DPIM. All the neurons in the network have the same architecture, which is shown in Figure 18. The hardware was developed only for the feedforward phase of MLP, adopting the weights defined in the training phase of the model in the software implementation, as described in SubSection 2.3.

This architecture has two submodules: the transfer function and the activation function. The neuron inputs are represented by $x_{1}$, $x_{2}$, …, $x_{m}$, where *m* is the number of inputs of the neuron specified. The $x_{0}$ represents the bias and it is set as a constant with a fixed value equal to −1. The output from the transfer function to the activation function is represented by $S_{k}$ and the neuron output is named $y_{k}$.

#### 3.2.1. Transfer Function Submodule

The Transfer Function Submodule is responsible for weighing the input of the neuron by their respective weights and summing these results, providing the output $S_{k}$ at the end.

**Figure 19:**
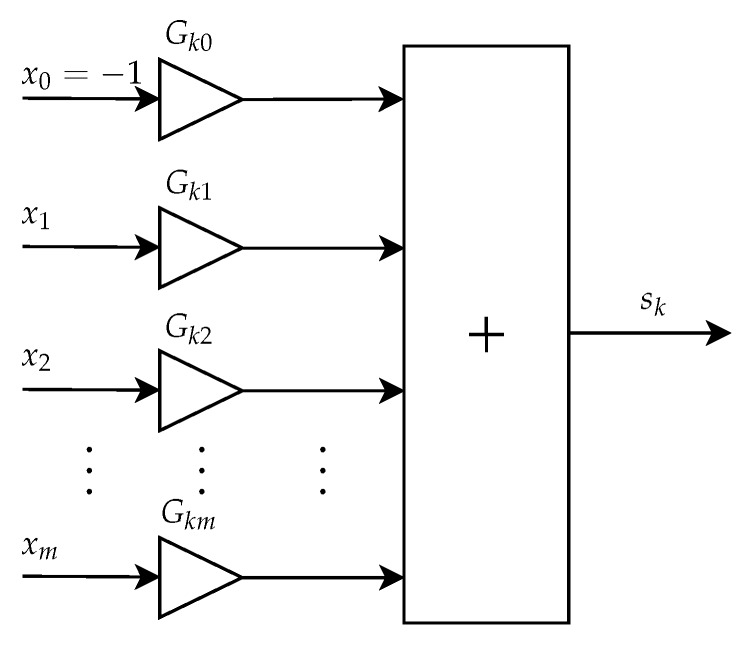
Architecture of the Transfer Function Submodule.

The architecture of this submodule is presented in Figure 19. The weighting of each neuron input is performed through a gain block configured with the value of the respective weight associated with the neuron input. The weights are represented by $W_{k0}$, $W_{k1}$, $W_{k2}$, …, $W_{km}$, where the first subscript term, *k*, indicates the neuron index and the second term indentifies the input to which the weight is associated. All the weights are fixed values represented as signed fractional numbers with 5 bits in the integer part and 10 bits in the fractional part. After the weighting of the inputs by their respective weights, all values obtained are summed, resulting in output $s_{k}$.

#### 3.2.2. Activation Function Submodule

Finally, the Activation Function Submodule is responsible for calculating the neuron output, $y_{k}$, based on the value provided by the Transfer Function Submodule.

**Figure 20:**
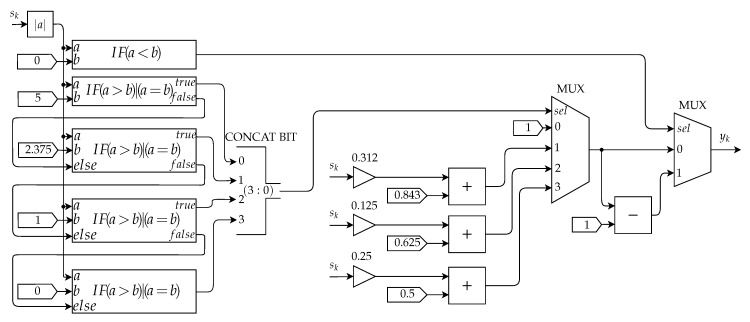
Sigmoide.

The architecture of this submodule is presented in Figure 20. The approximation of the Sigmoid function was performed using the PLAN [26] approximation method, which has the mathematical representation presented in Equation (13). Hence, the combinational blocks present in this architecture represent the conditions observed in this equation. Apart from the first conditional block from the architecture, the output of all other four conditional blocks are forwarded to the bus builder block, whose output selects in the MUX which equation is used to approximate the sigmoid value.

Some intervals of the approximated sigmoid are implemented by an adder which sums the value of a signal gain multiplied by the input of the sigmoid function to a constant from the equation expressed as

(13) $$f\left(x\right)=\left\{\begin{array}{cc} Y=1, & \mathrm{se}\left|x\right|\geq 5\\ Y=0.03125\cdot \left|x\right|+0.84375, & \mathrm{se}2.375\leq \left|x\right|<5\\ Y=0.125\cdot \left|x\right|+0.625, & \mathrm{se}1\leq \left|x\right|<2.375\\ Y=0.25\cdot \left|x\right|+0.5, & \mathrm{se}0\leq \left|x\right|<1\\ Y=1-Y, & \mathrm{se}x<0 \end{array}\right.$$

The first conditional block which refers to the last condition of Equation (13) analyzes whether the input value is less than zero. Its output is connected to the selective input of the last MUX block, which outputs b−1 if the $s_{k}$ is less than zero or *b* if it is greater than zero.

## 4. Results

This section presents the hardware validation results of the proposed implementation. The synthesis results present data associated with the use of hardware resources, execution time, and energy consumption. The implementation was synthesized on an Intel Cyclone V SE 5CSEBA6U23I7 FPGA, present in the DE10-Nano development kit. An ARM Cortex A-9 Dual-core 800MHz processor is also available in this kit. A video demonstration of the implementation is presented in [27].

### 4.1. Results from the Hardware Validation of the Implementation

The validation of the technique implemented in hardware was performed using the same validation images of the technique developed in software. The result obtained was identical to the equivalent software implementation, with the same correctness and errors in the classification of the images. As a consequence, the hardware technique obtained the same values of precision, specificity, and sensitivity presented in Table 1.

Thus, the system was validated showing that, although the proposed implementation in FPGA adopted fixed-point representation, the accuracy of the result was not compromised.

### 4.2. Synthesis Results

The results of the architecture synthesis enable the analysis of three different pieces of data: execution time, FPGA resource utilization, and energy consumption. Each of these analyses will be presented individually.

#### 4.2.1. Runtime

Initially, an implementation performance analysis related to execution time was conducted. For this, a comparison between the execution time of the implementation in the target FPGA, with a clock of 8.77 MHz, and the software equivalent implementation in the ARM Cortex A-9 Dual-core processor was performed. The execution time in the target FPGA was reached by multiplying the number of clock pulses required obtaining the results by the duration of a single clock. For the CPU, time was measured seven different times by a system function and the median value was used as the execution time.

First, the performance of the DPIM and ANNM modules were analyzed separately and, later, it was done for the complete architecture. The results presented in Table 2 were obtained by performing the execution time analysis of the DPIM in the FPGA and in the ARM processor. The obtained results point out that the DPIM FPGA implementation achieved better performance compared to the implementation in the ARM processor. This demonstrates the effectiveness of the implementation of DIP techniques in FPGA.

The results presented in Table 3 were obtained from the ANNM runtime analysis. A sample is the set of nine descriptors, which takes 0.6840μ s to be processed by the ANN implemented in FPGA, it achieved a throughput of approximately 1,461,988 samples per second. Thus, by comparing FPGA runtime with the processor, an increase in the processing speed of approximately 141.8167× was obtained.

According to the results collected after finishing the runtime analysis, the FPGA implementation was able to analyze 40 images per second, performing the analysis of 2414 images per minute. On the other hand, the ARM processor implementation is only capable of analyzing one image per second, resulting in 96 images per minute.

Given all the presented results, it is possible to affirm that the FPGA implementation generated a significant acceleration in the execution of the skin cancer detection technique.

The final analysis of the execution time is performed considering the complete system and the results presented in Table 4. According to the results collected after finishing the runtime analysis, the FPGA implementation was able to analyze 40 images per second, performing the analysis of 2414 images per minute. On the other hand, the ARM processor implementation is only capable of analyzing one image per second, resulting in 96 images per minute.

Given all the presented results, it is possible to affirm that the FPGA implementation generated a significant acceleration in the execution of the skin cancer detection technique.

#### 4.2.2. Hardware Resource Utilization

On top of the runtime analysis, another important performance parameter is the number of FPGA resources used. Here, again, a separate analysis for the DPIM and ANNM modules were conducted as well as an investigation for the hardware resources used by the complete implementation.

The use of hardware resources presented in Table 5 refers to the DPIM module. As can be seen from this table, the DPIM architecture used a low percentage of the hardware available in the FPGA.

The following Table 6 presents the hardware resources used by the ANNM module. Through the analysis of these results, it is possible to observe that, although the neural network implemented is relatively large, there are still enough free resources to implement it alongside the DPIM.

Finally, the resources used by the complete system architecture are presented in Table 7. Through it, it is possible to observe that the amount of resources used was low enough to allow the implementation of the architecture in the targeted FPGA. Based on these data, it is also possible to notice that the proposed implementation left more than 70% of the Adaptive Logic Modules (ALMs) available, which allows the implementation of additional logic.

#### 4.2.3. Power Consumption

The last performance parameter to be analyzed is energy consumption. This inspection was conducted using the Quartus Power Analyzer Tool for all inspected modules.

Following the same order as the previous analysis, here, the first module to be examined is the DPIM and the resulting power consumption is shown in Table 8.

Firstly, only the power consumption of the DPIM was analyzed. The results of the analysis are presented in Table 8.

Secondly, the same power consumption analysis was conducted for the ANNM module and the obtained results are shown in Table 9.

Finally, the result of the power consumption for the complete system is presented in Table 10.

The dynamic power consumption of the 800 MHz ARM processor is about 1392 mW while the FPGA implementation at a clock frequency of 8.77 MHz consumes about 159.06 mW. Hence, the FPGA saves 8.75× more power than the processor. It is also possible to reduce energy consumption even further by configuring the implementation to run with lower clock speeds.

### 4.3. State-of-the-Art Comparison

The implementation proposed in this work and presented in the previous sections will be compared to two other skin cancer detection systems from the literature: [14,15]. The comparison will be mainly focused on the ANNM model (Section 3.2), the module responsible for classification, which will be compared to the equivalent classifiers developed by the two mentioned references.

For comparison purposes, the models that obtained the best results in the studies of [14,15] were selected. Both developed classifiers based on SVM which were trained using a new 356 × 27 dataset (356 instances and 27 features) extracted from images collected from available web resources. In [14], this dataset was manipulated in order to generate two different datasets so that two modules could be produced: a melanoma-sensitive model called model M and non-melanoma-sensitive (benign) model called model N. These two models are then integrated into a cascade SVM system which is analyzed with and without the use of dynamic hardware technology. The dynamic partially reconfiguration (DPR) technique is exploited to allow dynamic reconfiguration of selected areas on FPGA on-the-fly. In [14], three models were created, but this comparison will only use the main two: Model 1, which used the original full dataset, and Model S, a smaller-scale implementation used as a case study.

The two models M and N proposed in [14] used 61 and 139 SVs respectively when implemented separately. The classifier implementation without dynamic hardware technology (Model 1 from [14]) used 200 SVs; in this, models M and N are simultaneously implemented on the hardware. Meanwhile, the dynamic hardware implementation (Model 2 from [14]) used 61 or 139 SVs, depending on the model, M or N, to be dynamically implemented in the hardware. In [15], their Model 1 was implemented with 248 SVs and the Model S was implemented with 61 SVs. For comparison, Design 1 of these two models proposed by [15] was used, which employed the optimization directive “Pipeline inner loops” of Vivado HLS.

#### 4.3.1. Runtime Comparison

Works [14,15] performed runtime measurement in two distinct ways. Due to the size limitation of the embedded DDR3 memory, the small-scale models [15] was used for evaluating the processing speed and time by using the XTimer IP measurements from running the application on Zynq Soc, while all other models were evaluated based on simulation and synthesis results.

Table 11 shows a comparison between the processing time results obtained by [14,15] and those obtained by the proposed work.

From this comparison, it can be seen that the classifier proposed in this work achieved an execution time of 0.68μs faster than the shortest time of 3μs reported by [14,15]. This difference represents a speedup of approximately 4.41×.

On the other hand, comparing the achieved results to the highest reported time of 141.38μs, a speedup of 208× was achieved.

#### 4.3.2. Hardware Resource Utilization

The target FPGA from [14,15] have LUTs that can be configured as either one 6-input LUT with one output or as two 5-input LUTs with separate outputs but common addresses or logic inputs. The FPGA used in the implementation proposed by the present work has the Adaptive Logic Module (ALM) as its basic element, composed of an 8-input fracturable look-up table (LUT) with four dedicated registers. For comparison, it is going to be assumed that 1 ALM is equivalent to 1 LUT from the target [14,15]. The same frequencies from Table 11 were used for the hardware synthesis perfmored in order to generate the presented results regarding resources usage.

Table 12 shows a comparison between the results obtained by [14,15] and those obtained by the proposed work.

From the above results, it can be seen that the ANNM classifier presented a higher usage of ALMs and DSPs in relation to the equivalent usage from the other models. The opposite occurred for the number of registers. This happens because in this case there is the implementation of two distinct classifiers, the SVM and the MLP. The MLP performs a greater number of operations, each neuron has a multiplication for each of its inputs, a cascade sum of these results and an approximation of the activation function through sums, multiplications, and other logical components. While the SVM requires a smaller number of operations to obtain its classification, there is basically one multiplication by SV and cascade sum.

#### 4.3.3. Power Consumption Comparison

Ref. [14,15] used the Vivado tool for power consumption analysis of all implementations. Table 13 shows a comparison between the power consumption results obtained by [14,15] and those obtained by the proposed ANNM.

From the above results, it can be noticed that the proposed ANNM hardware implementation achieved the lowest dynamic power consumption and the highest static power consumption among all the compared implementations. Overall, the proposed ANNM achieved the lowest total energy consumption of 0.5177 W which represents a reduction of approximately 3× if compared to the lowest power consumption, 1.550 W [14], and a reduction of approximately 3.4× if compared to the highest power consumption observed, 1.756 W [15].

#### 4.3.4. Accuracy Comparison

Finally, the accuracy results obtained in this work are compared with the models [14,15], as shown in Table 14.

The models proposed in [14,15] were trained using cross-validation, and the accuracy result presented for each model is the the cross-validation accuracy. This accuracy is obtained with the same set of data that is used in training at a given moment, generally providing greater accuracy than that obtained with validation data not used in training, while, in this work, the dataset was divided into training and validation data, to obtain accuracy using only validation data not used in training. Sensitivity and specificity results are not compared as these data are not presented by [14,15].

The FPGA implementation of cascade SVMs [14] used a melanoma-sensitive model and a non-melanoma-sensitive model, with accuracies of 97.92% and 72,51%, respectively; therefore, both accuracies are reported in Table 14. From Table 14, the model here presented obtained a accuracy of 90% in the validation data, this result is better than the Model 1 [15] and the non-melanoma-sensitive model presented in the cascade SVMs from [14]. The proposed model shows inferior results to the model S [15] and the melanoma-sensitive model in the cascade SVMs from [14]. Although our result is lower than these, there is a problem with the different methods of determining accuracy. The models proposed in [14,15] can present better results than what would be obtained by adopting the same method of determining accuracy used in this work. Moreover, this work uses a different database than the one used in the comparative works, which can also influence the accuracy obtained.

## 5. Conclusions

This work implemented a detection system of skin cancer based on reconfigurable hardware using FPGA based on artificial neural networks and digital image processing techniques. The Hardware architecture was developed using fixed-point representation. When comparing the hardware implementation to a software implementation using float-point precision, the same values of accuracy, sensitivity, and specificity were obtained. This means that, even with a reduced numerical precision, it was possible to maintain the same statistical result while saving enough hardware resources for additional logic to be implemented if needed in the Intel Cyclone V SE 5CSEBA6U23I7 FPGA.

The implementation of the DPI and MLP neural network techniques in FPGA showed better results than the respective implementations in the ARM processor, achieving better runtime and lower power consumption. The execution time of the complete hardware system was approximately 25× faster than the equivalent software implementation. The FPGA implementation required about 5× less power than the processor. Compared to other similar works in the literature, the implementation proposed here presented achieved a runtime up to 208× faster, low hardware resource utilization, power consumption up to 3.4× lower, and an accuracy value better than a few models and in the same range of precision of other models.

For future work, the authors from the presented implementation plan to keep working in the classification phase in order to improve the overall classification accuracy of the system and perform new tests with larger datasets. In addition to that, it is possible to design a sub-module in the Digital Image Processing Module to automatically generate the mask, which would significantly increase parallelism in the implementation.

## Figures and Tables

**Table 1:** Comparison between the proposed method and works from literature.

Method	Accuracy	Specificity	Sensitivity
[21]	82.6%	89.8%	53.3%
[22]	81%	80%	81%
[23]	87.8%	97.9%	78.4%
[24]	85.5%	93.1%	54.7%
**This work**	90%	90.9%	87.5%

**Table 2:** DPIM runtime comparison.

Data	Dual-Core ARM	FPGA
**Runtime (s)**	0.6227	0.0245
**Images p/second**	1.6059	40.8163
**Speedup**	1	25.4165

**Table 3:** ANNM runtime.

Data	Dual-Core ARM	FPGA
**Runtime (** μ **s)**	97	0.6840
**Samples p/second**	10,309	1,461,988
**Speedup**	1	141.8167

**Table 4:** Runtime of the complete system.

Data	Dual-Core ARM	FPGA
**Runtime (s)**	0.6228	0.0245
**Samples p/second**	1.6057	40.8163
**Speedup**	1	25.4165

**Table 5:** Hardware resources used by the DPIM Module.

Resources	Total
**ALMs**	3207 (8%)
**Pins**	133 (42%)
**Bits of Memory Blocks**	256 (<1%)
**DSP blocks**	4 (4%)
**Registers**	459 (<1%)

**Table 6:** Hardware resources used by the ANNM Module.

Resources	Total
**ALMs**	6684 (16%)
**Pins**	123 (39%)
**Bits of Memory Blocks**	0 (0%)
**DSP blocks**	112 (100%)
**Registers**	1482 (<1%)

**Table 7:** Hardware Resourcers used by the entire implementation.

Resources	Total
**ALMs**	9645 (23%)
**Pins**	56 (18%)
**Bits of Memory Blocks**	256 (<1%)
**DSP blocks**	112 (100%)
**Registers**	2067 (<1%)

**Table 8:** Power consumption for the DPIM module.

Power Consumption	Total (mW)
**Dynamic**	43.85
**Static**	412.69
**I/O**	14.23
**Total**	470.77

**Table 9:** Power consumption for the ANNM module.

Power Consumption	Total (mW)
**Dynamic**	104.32
**Static**	413.36
**I/O**	42.56
**Total**	560.24

**Table 10:** Power consumption for the entire implementation.

Power Consumption	Total (mW)
**Dynamic**	159.06
**Static**	413.89
**I/O**	41.39
**Total**	614.34

**Table 11:** Runtime Comparison with related works.

Related Works	Model 1 [14]	Model 2 [14]	Model 1 [15]	Model S [15]	Proposed ANNM
**Frequency (MHz)**	100	100	100	250	8.77
**Runtime (μs)**	3	3	141.38	14.77	0.68

**Table 12:** Comparison of Hardware Resources usage with related works.

Related Works	Model 1 [14]	Model 2 [14]	Model 1 [15]	Model S [15]	Proposed ANNM
**ALMs/LUTs**	1762	1457	2579	2870	6684
**DSP**	10	5	5	5	112
**Registers**	N/A	N/A	2898	3332	1482

**Table 13:** Comparison of power consumption with related works.

Related Works	Model 1 [14]	Model 2 [14]	Model 1 [15]	Model S [15]	Proposed ANNM
**Dynamic (W)**	1.4040	1.3950	1.5980	1.5343	0.1043
**Static (W)**	0.1560	0.1550	0.1580	0.1517	0.4134
**Total (W)**	1.5600	1.5500	1.7560	1.6860	0.5177

**Table 14:** Comparison of classification accuracy with related works.

Related Works	Model 1 [14]	Model 2 [14]	Model 1 [15]	Model S [15]	Proposed ANNM
**Accuracy**	97.9% and 72.5%	97.9% and 72.5%	80.85%	97.92%	90%
